# Development of a Machine Learning Classifier Based on Radiomic Features Extracted From Post-Contrast 3D T1-Weighted MR Images to Distinguish Glioblastoma From Solitary Brain Metastasis

**DOI:** 10.3389/fonc.2021.638262

**Published:** 2021-07-13

**Authors:** Alix de Causans, Alexandre Carré, Alexandre Roux, Arnault Tauziède-Espariat, Samy Ammari, Edouard Dezamis, Frederic Dhermain, Sylvain Reuzé, Eric Deutsch, Catherine Oppenheim, Pascale Varlet, Johan Pallud, Myriam Edjlali, Charlotte Robert

**Affiliations:** ^1^ Neuroradiology Department, Hôpital Sainte-Anne, GHU-Paris Psychiatrie et Neurosciences, Paris, France; ^2^ Université de Paris, Paris, France; ^3^ Inserm, UMR1266, IMA-Brain, Institut de Psychiatrie et Neurosciences, Paris, France; ^4^ Radiothérapie Moléculaire et Innovation Thérapeutique, INSERM UMR1030, Gustave Roussy Cancer Campus, Université Paris Saclay, Villejuif, France; ^5^ Département de Radiothérapie, Gustave Roussy, Université Paris Saclay, Villejuif, France; ^6^ Service de Neurochirurgie, GHU Paris – Psychiatrie et Neurosciences – Hôpital Sainte-Anne, Paris, France; ^7^ Service de Neuropathologie, GHU Paris – Psychiatrie et Neurosciences – Hôpital Sainte-Anne, Paris, France; ^8^ Département de Radiologie, Gustave Roussy, Université Paris Saclay, Villejuif, France; ^9^ BioMaps UMR1281, Université Paris-Saclay, CNRS, INSERM, CEA, Orsay, France

**Keywords:** radiomics, machine learning, glioblastoma, brain metastasis, diagnostic decision support system

## Abstract

**Objectives:**

To differentiate Glioblastomas (GBM) and Brain Metastases (BM) using a radiomic features-based Machine Learning (ML) classifier trained from post-contrast three-dimensional T1-weighted (post-contrast 3DT1) MR imaging, and compare its performance in medical diagnosis *versus* human experts, on a testing cohort.

**Methods:**

We enrolled 143 patients (71 GBM and 72 BM) in a retrospective bicentric study from January 2010 to May 2019 to train the classifier. Post-contrast 3DT1 MR images were performed on a 3-Tesla MR unit and 100 radiomic features were extracted. Selection and optimization of the Machine Learning (ML) classifier was performed using a nested cross-validation. Sensitivity, specificity, balanced accuracy, and area under the receiver operating characteristic curve (AUC) were calculated as performance metrics. The model final performance was cross-validated, then evaluated on a test set of 37 patients, and compared to human blind reading using a McNemar’s test.

**Results:**

The ML classifier had a mean [95% confidence interval] sensitivity of 85% [77; 94], a specificity of 87% [78; 97], a balanced accuracy of 86% [80; 92], and an AUC of 92% [87; 97] with cross-validation. Sensitivity, specificity, balanced accuracy and AUC were equal to 75, 86, 80 and 85% on the test set. Sphericity 3D radiomic index highlighted the highest coefficient in the logistic regression model. There were no statistical significant differences observed between the performance of the classifier and the experts’ blinded examination.

**Conclusions:**

The proposed diagnostic support system based on radiomic features extracted from post-contrast 3DT1 MR images helps in differentiating solitary BM from GBM with high diagnosis performance and generalizability.

## Introduction

Brain Metastases (BM) and Glioblastomas (GBM) are the two most frequent intra-cranial brain tumors in adults (1–3). Currently, Magnetic Resonance Imaging (MRI) is the modality of choice for brain tumor characterization. Usually, BM present an encapsulated contrast enhancement, with regular and well-defined boundaries, whereas GBM have heterogeneous contrast enhancement with very irregular and fuzzy boundaries (4–6). Nonetheless, their morphological characteristics remain very similar on MRI as both are lesions with annular contrast enhancement, having a necrotic center and a peritumoral zone in T2-weighted and Fluid-Attenuated Inversion Recovery (FLAIR) sequences. Advanced neuroimaging techniques such as perfusion MRI and Magnetic Resonance Spectroscopy (MRS) provide additional information to distinguish between the two tumor types, based on differences in the peritumoral area (7–10). Although in the past decades, various studies (11–13) have evaluated the diagnostic performance of perfusion imaging and MRS, they have shown heterogeneous results in distinguishing these two tumor types, resulting in sensitivities and specificities ranging from 64 to 100% and 60 to 100% respectively. This high heterogeneity reflects the difficulty experienced in daily practice to differentiate the two brain tumors, even using advanced neuroimaging techniques, particularly in the case of differentiating a GBM from a solitary BM revealing an unknown primary cancer [5 to 12% of BM (14, 15)]. Even though the final diagnostic will be given by a histopathological examination and a biomolecular analysis of the tumor tissue relying on the 2016 WHO classification (16), the presurgical distinction between these two types of tumors is crucial for adapting treatment strategies: for metastases less than 3–4 cm, a bloc resection or stereotactic radiosurgery will be planned depending on the lesion location (17), while GBM (18) should be treated with maximal safe resection, and concurrent chemoradiotherapy. Radiomics (19–22) is a recent area of research based on the simple observation that the human eyes have limitations, even those trained for medical image interpretation. Radiomics consists of extracting large numbers of predefined quantitative features from medical images with the ultimate goal of identifying subgroups of biomarkers able to guide patient’s care and has shown promise in brain cancer detection, diagnosis, molecular mutation characterization, prognosis and outcome prediction (23–29). In our study, we hypothesized that the morphological differences observed on post-contrast 3DT1 MR images would lead to differences in radiomic features between the two tumor types. The aim of this study was to therefore develop a radiomic features-based Machine Learning (ML) classifier, to evaluate its diagnostic performance on an unseen test set of patients, and to compare it to the diagnosis performance of neuroradiologists. A strong emphasis was placed on favoring explainable classifiers to ease translation into clinic.

## Materials and Methods

**Figure 1:**
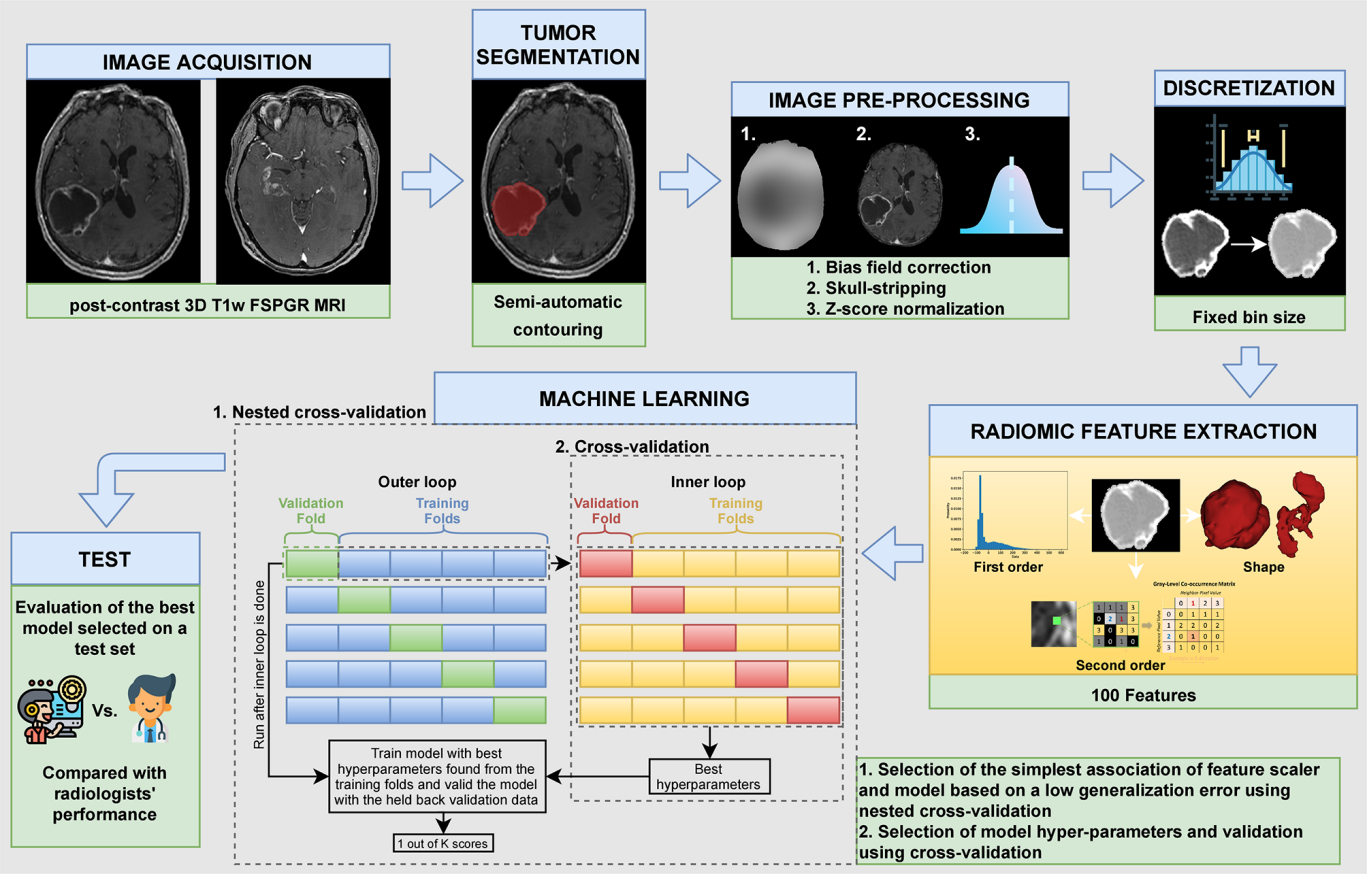
Different steps of the study.

The steps of our study are summarized in **Figure 1**.

### Patients

This retrospective bicentric study was approved by the local institutional review board (n° IRB00011687 College de neurochirurgie IRB #1: 2020/29). The two Radiology Departments that participated in the study had the same 3 Tesla MRI scanners (MR 750, Discovery; General Electric Healthcare), with the same imaging parameters implemented. Medical records of patients who had histologically proven BM or GBM between January 2010 and May 2019 were screened in the two centers to constitute the training set. Inclusion criteria for the training set were: 1) patients more than 18 years of age, 2) with histologically-confirmed diagnosis of BM or GBM, and 3) and with pre-operative MRI. Exclusion criteria for the training set were: 1) lesions less than 2 cm, 2) extra-axial locations, 3) history of treatment before the MRI examination, 4) absence of 3D T1-weighted Fast SPoiled Gradient Recalled sequence, 5) image acquisition performed on a different machine to the 3 Tesla GE Discovery MR scanner, and 6) 3D T1-weighted sequence acquired with non-conventional parameters or inadequate quality (see section *MRI data*). The minimal size of 2 cm was chosen as GBM are usually >2 cm at the diagnosis. We therefore wanted to exclude small BM from the analysis, to avoid a bias of size. For BM, we included patients with one or more brain lesions. However in cases of multiple lesions, only the largest was segmented for radiomic feature extraction.

**Figure 2:**
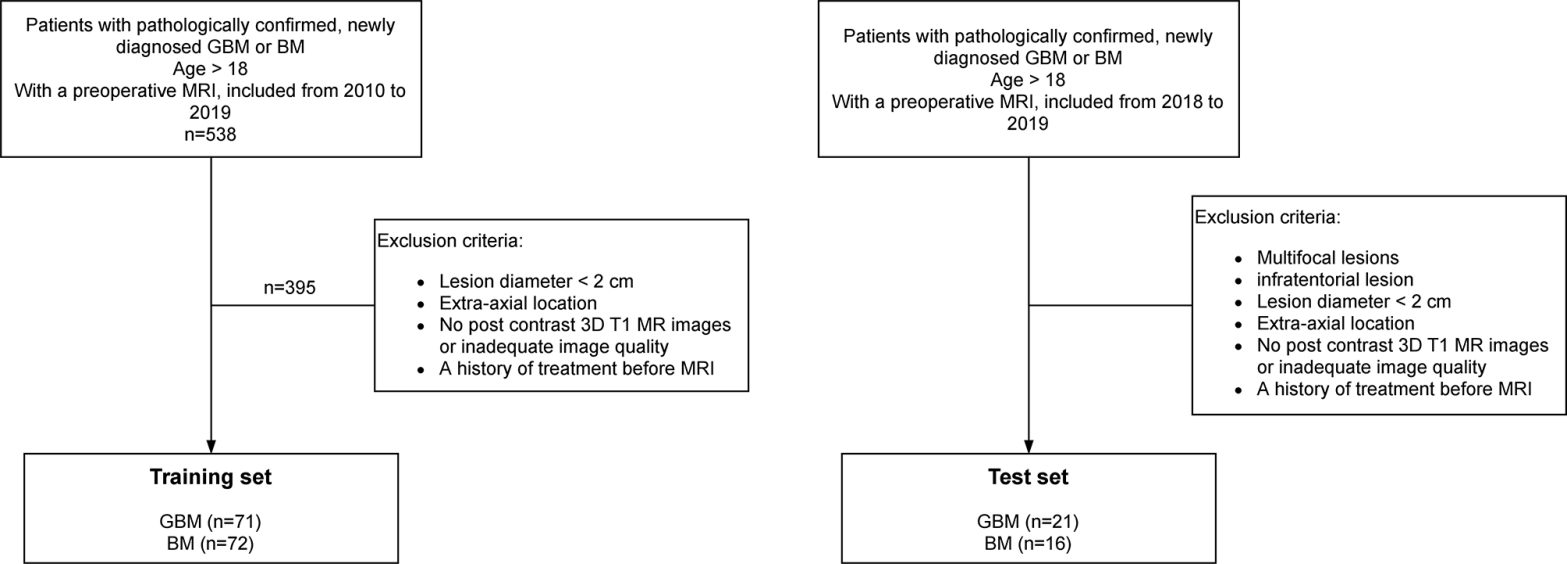
Flow chart of patient inclusion.

Secondly, a test set was constituted after completion of the model development process in order to evaluate the final performance of the radiomic classifier on unseen lesions. As well, the test set included patients from both centers. Inclusion criteria for the test set were the same as for the training set. All patients included in the test set were required to have solitary lesions so that neuroradiologists were not influenced in their final diagnosis. Exclusion criteria of the study were therefore the same as those of the training set plus patients having multifocal or infra-tentorial lesions. All inclusion and exclusion criteria are summarized in the flow chart (**Figure 2**).

### MRI Data

MR acquisitions were performed on the same 3 Tesla MR scanner, even if at two clinical sites. MRI data included at least a post-contrast (gadoterate meglumine [Dotarem; Guerbet Laboratory]) three-dimensional T1-weighed Fast SPoiled Gradient Recalled (FSPGR) acquisition (post-contrast 3DT1), with the following parameters: repetition time: 10.2 ms; echo time: 3.4 ms; field of view: 22 cm; voxel size: 0.8 mm × 0.8 mm × 1.2 mm. Patients were excluded from this study if other imaging protocols were followed. Post-contrast 3DT1 MR images were only used as inputs of the radiomic classifier. To compare the performance between the classifier and neuroradiologists, clinical conditions were mimicked, and all available sequences of the imaging exam were thus analyzed by the neuroradiologists, as routinely conducted in a clinical setting.

### Image Analysis

#### Pre-Processing

MR image preprocessing included bias field correction using the N4ITK algorithm (30) from the Advanced Normalization Tools (ANTs) library (31), skull-stripping with the Brain Extraction Tool (BET) of the FSL software (FMRIB’s Software Library) (32) and Z-score normalization with a scaling factor of 100. No spatial resampling was performed due to data homogeneity. As well, no noise filtering was applied.

#### Tumor Segmentation

A segmentation of the volume of interest, including the contrast-enhanced and necrotic regions, was performed semi-automatically using Olea Sphere^©^ (Olea Medical, La Ciotat, France). These two sub-regions corresponded to Labels 4 and 1 of the BraTS 2012–2016 challenge (33). Within a region of interest defined by a trained radiologist (AdC, 5 years of experience), threshold-based gray level contouring and manual correction were used for the segmentation so that the volume of interest was carefully drawn along the tumor enhancement.

#### Feature Extraction

One hundred radiomic features were extracted from the 3D MR images using the Python library PyRadiomics 2.1.2 (34) in which the feature definitions are consistent with the Image Biomarker Standardization Initiative (IBSI) (35). The only exception is that PyRadiomics and IBSI use different definitions of the Kurtosis first-order feature, where Kurtosis is calculated using −3 and +3 in the IBSI and PyRadiomics referentials respectively. For first order features, an intensity shifting of 300 (equal to three standard deviations) was applied to ensure that the majority of the voxel intensities were positive before feature extraction. An absolute discretization with a fixed bin size equal to 37 was chosen (36, 37). This leads to a bin number of 32 considering the mean of the intensity intervals computed for all volumes of interest of patients of the training set (min intensity: 575, max intensity: 2069, mean intensity range: 1190). Six feature classes were considered: 18 first-order statistics, 14 shape-based features, 22 Gray Level Co-occurrence Matrix features (GLCM), 16 Gray Level Run Length Matrix features (GLRLM), 16 Gray Level Size Zone Matrix features (GLSZM), and 14 Gray Level Dependence Matrix features (GLDM).

### Model Building

The establishment of the classification model was based on the scikit-learn library version 0.23.2 (38) and included two steps applied to the training set: (1) selection of the ML classifier and feature scaling method and 2) optimization of the hyper-parameters. In step 1), a nested cross-validation was used given the moderately-sized dataset and 144 ML models combining nine feature scaling methods (No Scaler, MaxAbsScaler, MinMaxScaler, Normalizer, PowerTransformer-Yeo–Johnson, QuantileTransformer-normal, QuantileTransformer-uniform, RobustScaler, StandardScaler) and 16 classifiers (AdaBoostClassifier, BaggingClassifier, BernoulliNB, DecisionTreeClassifier, ExtraTreeClassifier, ExtraTreesClassifier, GaussianNB, GradientBoostingClassifier, KNeighborsClassifier, LinearSVC, LogisticRegression, MLPClassifier, QuadraticDiscriminantAnalysis, RandomForestClassifier, RidgeClassifier, SGDClassifier) were compared. The nested cross-validation considered a stratified 5-fold cross-validation in the inner loop for hyper-parameter tuning (grid search strategy) and a stratified 5-fold cross-validation in the outer loop for the evaluation of the performance of the model. In step 2), the model showing the lowest generalization error, as assessed by the balanced accuracy, was kept and a ten-repeated 5-fold cross-validation was performed. In this second step, a grid search method was implemented to optimize the final set of hyper-parameters. Mean sensitivity, specificity, balanced accuracy, and area under the receiver operating characteristic curve (AUC) and their associated variances and 95% confidence intervals were calculated as performance metrics. Research spaces for hyper-parameter tuning with grid search during nested cross-validation and cross-validation are described in **Table S1**.

### Evaluation on the Test Set and Comparison to Human Performance

The final model was fitted using the entire training set and its performance evaluated on the test set including 37 patients (21 GBM and 16 BM). Images of the test set were then blindly analyzed by five neuroradiologists (R1, R2, R3, R4, and R5). Two were neuroradiologists with more than 10 years of experience and three were radiology residents with about 6 months of training and practice in neuroradiology. The neuroradiologists had access to all MR sequences acquired in a routine MR imaging protocol, including 3D FLAIR, 2D T2, perfusion imaging, and pre- and post-contrast 3DT1 sequences.

### Statistics

Sensitivity, specificity, balanced accuracy and AUC were used to assess the diagnosis performance of the radiomic model. We applied a McNemar’s test and evaluated its p-value to assess if the differences were significant between the diagnostic performance of the radiomic classifier and the diagnostic performance of the readers. The threshold was set at 0.05.

## Results

### Patients

267 GBM and 271 BM were pre-selected for the training set, and 71 GBM and 72 BM met the inclusion criteria respectively (**Figure 2**). Median [minimum value–maximum value] 2D maximal diameter was equal to 53.39 mm [24.11–88.12 mm] for GBM and 41.40 mm [20.77–77.92 mm] for BM. The test set included 37 patients (21 GBM and 16 BM). In this set, the median 2D maximal diameter was equal to 54.93 mm [32.61–102.53 mm] and 33.85 mm [22.41–63.63 mm] for GBM and BM respectively. Patient characteristics and their repartition between Centers 1 and 2 are summarized in **Table 1**.

**Table 1:** Demographics and clinical characteristics at diagnosis of the patients included in the training set and in the test set.

Patients characteristics	Training set		Test set	
	BM (n = 72)	GBM (n = 71)	BM (n = 16)	GBM (n = 21)
Mean patient age—years	59.29	58.25	59.00	58.19
(standard deviation)	(13.29)	(14.59)	(10.9)	(14.5)
Proportion of female gender (%)	53	38	50	52
Proportion of male gender (%)	47	62	50	48
Largest diameter in mm	41.40	53.39	33.85	54.93
median [range]	[20.77–77.92]	[24.11–88.12]	[22.41–63.63]	[32.61–102.53]
Patients from Center 1	56 (77.8%)	69 (97.2%)	5 (31.2%)	18 (85.7%)
Patients from Center 2	16 (22.2%)	2 (2.8%)	5 (31.2%)	3 (14.3%)
Primary lung cancer n (%)	29 (40.3)	–	8 (50)	–
Primary breast cancer n (%)	13 (18.0)	–	3 (18)	–
Melanoma n (%)	9 (12.5)	–	2 (12.5)	–
Primary colo-rectal cancer n (%)	5 (6.9)	–	0 (0)	–
Primary Clair cell carcinoma n (%)	4 (5.6)	–	1 (6.3)	–
Other primary cancer * n (%)	12 (16.7)	–	2 (12.5)	–

*Primary rare cancer: choriocarcinoma, sarcoma, salivary gland carcinoma, papillary carcinoma of the thyroid.

### Selected Machine Learning Classifier

**Figure 3:**
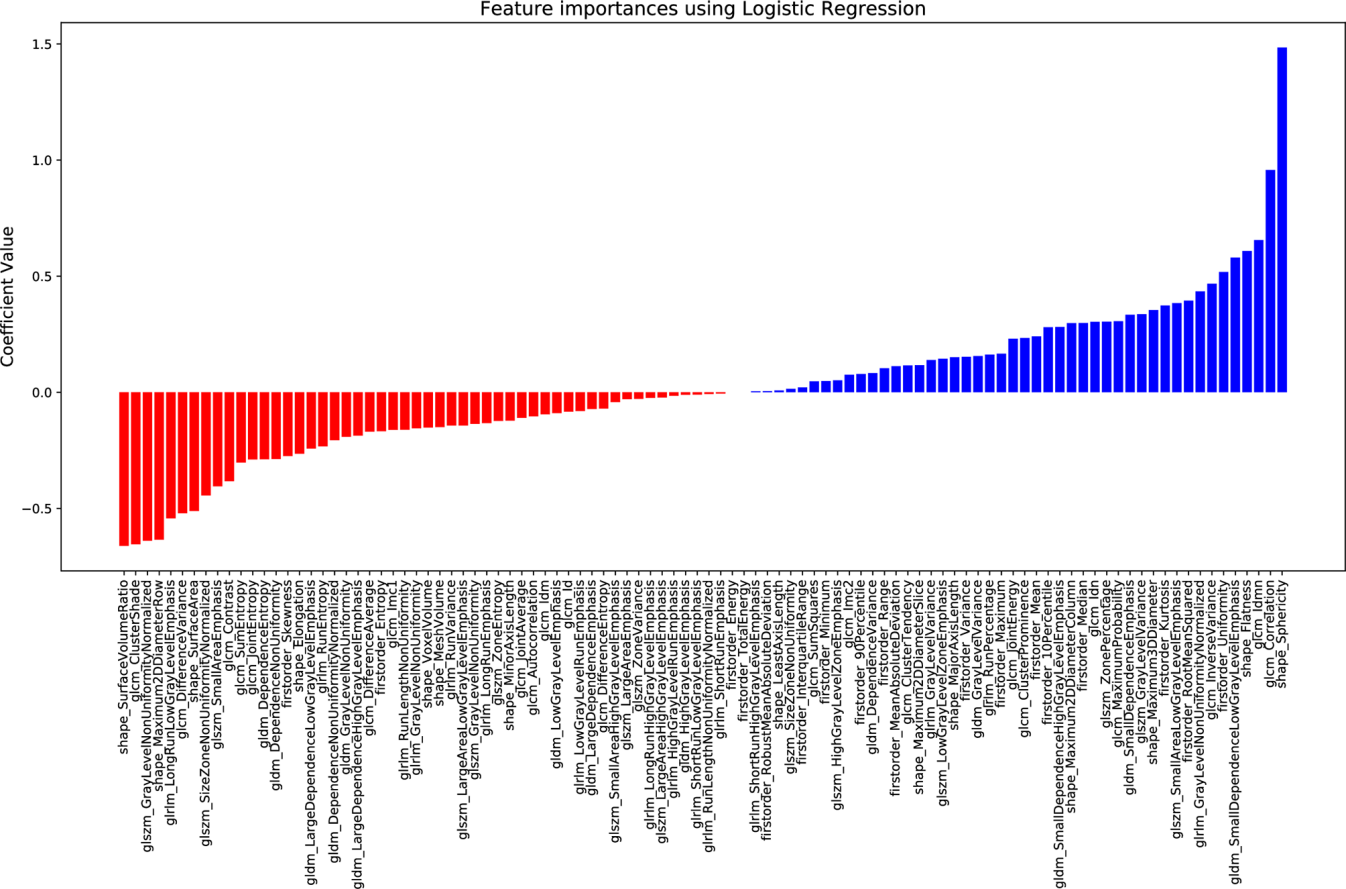
Coefficient of each radiomic feature in the decision function for the proposed logistic regression model.

**Table S2** summarizes the mean balanced accuracies and their associated standard deviations obtained for all tested combinations (scaling method + classifier). Combinations are ranked considering the lowest generalization error. The ML classifier providing the better performance using the nested cross-validation was the logistic regression combined to the power transform Yeo–Johnson scaling feature method which corresponds to a zero-mean, unit-variance normalization with a power transform applied feature wise to make distribution of each radiomic feature Gaussian-like. To limit overfitting, the classifier encompassed a ridge regression for regularization (l2 penalty assignment) with a C value equal to 0.7. The final logistic regression-based established signature was a combination of the 100 input radiomics features, in which the feature with the highest coefficient in the decision function was sphericity, with a coefficient of 1.48. All other features had absolute coefficient less than 0.96. The 20 predominant features had absolute coefficients superior to 0.38. Among these features, five were shape features, two were first-order metrics, and 13 were based on texture matrices, with 6 extracted from the GLCM matrix (**Figure 3**).

### Diagnosis Performance of the Classifier With a Ten-Repeated 5-Fold Cross-Validation

**Figure 4:**
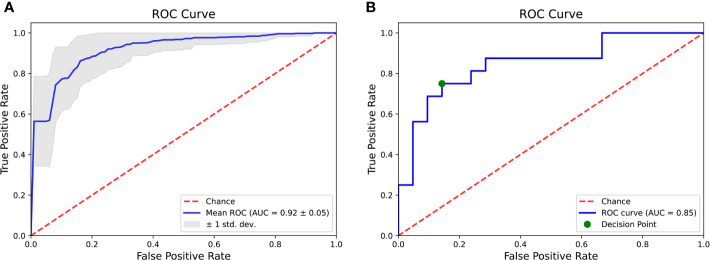
Areas under the receiver operating characteristics curve of the radiomic classifier after ten-repeated 5-fold cross-validation **(A)** and on the test set **(B)**.

The model differentiated BM from GBM on the validation sets with a mean sensitivity of 85% [95% CI = (77%; 94%)], a specificity of 87% [95% CI = (78%; 97%)], a balanced accuracy of 86% [95% CI = (80%; 92%)], and an AUC of 92% [95% CI = (87%; 97%)] (**Figure 4**).

### Diagnosis Performance of the Radiomic Classifier on the Test Set

**Figure 5:**
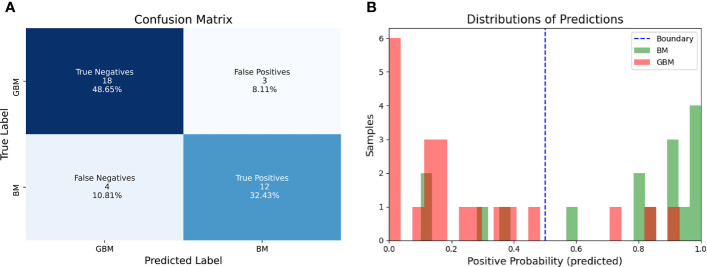
Confusion Matrix of the radiomic model on the test set **(A)** and distribution of probabilities as predicted by the logistic regression model compared to ground truth **(B)**.

The classifier correctly identified 12/16 BM and 18/21 GBM. Corresponding sensitivity, specificity, balanced accuracy and AUC were respectively equal to 75, 86, 80, and 85% (**Figures 4** and **5**).

### Performance of the Radiologists

The performances of the neuroradiologists are described in **Table 2**. Even though differences in diagnostic performance were not statistically significant, we can highlight the fact that two radiology residents (R3 and R4) had lower scores than the classifier (respective balanced accuracies of 72 and 72%) whereas the two neuroradiologists with 10 years of experience (R1 and R2) and one radiology resident (R5) had better scores than the classifier (respective balanced accuracies of 87, 94 and 88% *versus* balanced accuracy of 80% for the classifier).

**Table 2:** Sensitivities, specificities, balanced accuracies, positive predictive values, negative predictive values of the radiomic classifier and of the neuroradiologists (R1, R2, R3, R4, R5) on the test set.

Reader	Se*	Sp*	Balanced Accuracy	PPV*	PNV*	Se p-value*	Sp p-value*
Radiomic classifier	0.75	0.86	0.8	0.8	0.82	–	–
R1	0.88	0.86	0.87	0.82	0.9	0.41	1
R2	0.94	0.95	0.94	0.94	0.95	0.08	0.16
R3	0.69	0.76	0.72	0.76	0.69	0.65	0.41
R4	0.63	0.81	0.72	0.71	0.74	0.48	0.65
R5	0.81	0.95	0.88	0.93	0.87	0.65	0.16

*Se, Sensitivity; Sp, Specificity; PPV, Positive Predictive Value; PNV, Positive Negative Value; Se p-value, p-value (calculated with McNemar’s test) of the difference between the sensibility of the radiomic classifier and the sensibility of the reader; Sp p-value, p-value (calculated with McNemar’s test) of the difference between the specificity of the radiomic classifier and the specificity of the reader.

## Discussion

We have developed a radiomic classifier to differentiate solitary BM and GBM based on post-contrast 3DT1 MR images with high diagnostic performances on the validation and test sets. There was no statistically significant difference between classifier predictions and human reading by five trained neuroradiologists (two neuroradiologists with 10 years of experience, and three radiology residents with about 6 months of training exclusively in neuroradiology in an expert center).

**Figure 6:**
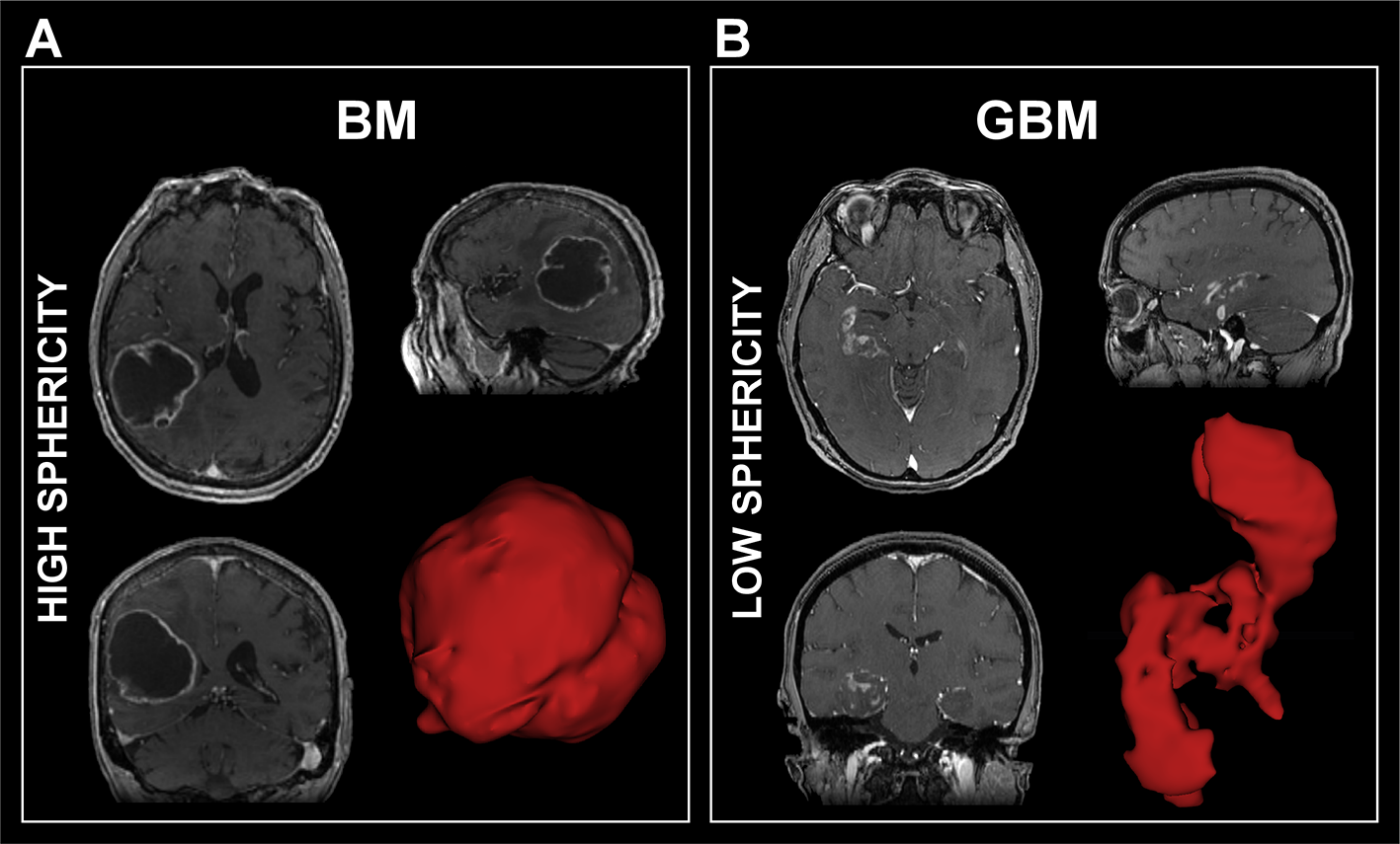
Examples of 3D representation of a brain metastasis **(A)** for which the sphericity was equal to 0.76 and a glioblastoma **(B)** for which the sphericity was equal to 0.45. GBM, Glioblastoma; BM, Brain Metastasis.

The radiomic classifier, a logistic regression combined to the power transform Yeo–Johnson scaling feature method, was chosen because of its high performance, simplicity, and because it allowed an interpretation of the underlying model. Indeed, the fact that the radiomic feature with the most important coefficient value in the classifier was a shape feature, i.e. sphericity, partly allows an explainability of our radiomic features-based classifier in contrast with the concept of the “black box” in some ML models, where even its designers cannot explain why the artificial intelligence reaches a decision (39). It introduces the notion of analyzing a tumor with its representation in 3D to differentiate solitary BM and GBM, which is usually not available during conventional reading of sectional imaging. Indeed, sphericity is a 3D shape feature representing a measure of roundness of the tumor, with a value ranging from 0 to 1, where 1 indicates a perfect sphere. The classifier showed that GBM have lower sphericity than BM (**Figure 6**), which was expected given the morphological characteristics of BM and GBM on histopathological slides. The more spherical the lesion is, the more likely it is to be a BM. Thus, the radiomic features-based classifier is consistent with current morphological characteristics between BM and GBM, also adding further information regarding tumor heterogeneity imperceptible to the human eye, as the radiomic classifier is also based on other texture and intensity features. This result is in line with a pioneering paper (40) that described in 2012 2D circularity as one of the best morphological features to differentiate BM from GBM on the basis of a cohort of 50 patients.

**Figure 7:**
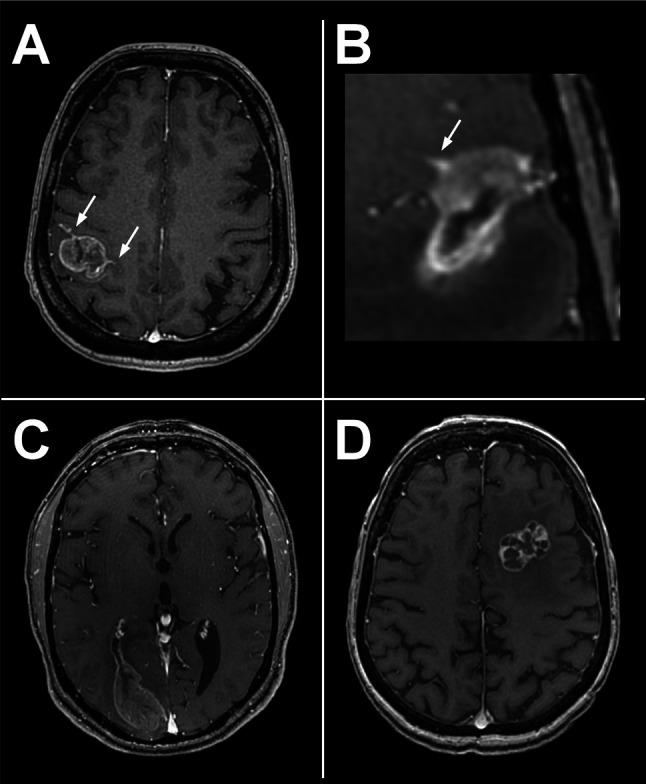
Four incorrectly classified BM of the test set. Two of them presented tumoral leptomeningitis (arrows, **A, B**), one a metastatic ventriculitis **(C)** and the forth one a multilocular lesion **(D)**. Leptomeningitis and ventriculitis may have interfered with spatial delineation of tumor boundaries.

In our study, we trained the ML classifier using a nested cross-validation and a ten-repeated 5-fold cross-validation on the training set in order to minimize overfitting. In addition to limit the extraction to 100 features (shape, first order and second order features) that we thought to be the most meaningful and interpretable, we selected a classifier model which could embed feature selection. For this model, L1 and L2 regularization methods were tested as hyperparameters. The L2 method provided the best performance in the cross-validation (CV) process, validating the usefulness of the 100 features. The selected classifier was then applied on a test set of data, which demonstrates that the high performances obtained were not random but generalizable. In the test set, 12/16 BM were correctly classified leading to a sensitivity of 75%. Among the four BM incorrectly classified, two had leptomeningeal enhancement, one had ventriculitis adjacent to the lesion and the fourth one had a multilocular lesion (**Figure 7**). The first three elements were absent from BM of the training set, which might have misled the classifier, suggesting the need for a larger training set which extensively reproduces all clinical situations encountered in clinic.

The results of our study are consistent with the results of three previous studies which also used radiomic features-based classifiers on post-contrast 3D T1 MR images to differentiate BM from GBM. Among these studies, Chen et al. (41) achieved diagnostic performance slightly lower than our on 134 patients, however without applying image pre-processing (42–44) nor evaluation on a test set. Artzi et al. (45) built a radiomics-based classifier on 358 patients and evaluated its performance on a test set of 88 patients. Excellent performances were achieved on the test set. However, the radiomic analysis was carried out on three central slices only to simplify the segmentation process, which did not allow 3D shape features such as sphericity, to be taken into account. Moreover, there was no comparison to human performance. In 2019, Qian et al. (46) used a cohort of 227 patients to train a ML classifier using cross-validation and evaluated it on an independent test set of 185 patients. Despite high diagnostic performances, there were biases in the study considering several radiomic features-based classifiers were evaluated on the test set. Finally, in 2020, Bae et al. (47) developed a Deep Neural Network (DNN) classifier based on post-contrast 3D T1-weighted and T2-weighted MR images, which outperformed the best-performing traditional machine learning model. Results showed excellent performance on an independent test set (AUC of 0.956 on the test set) and outperformed scores of two trained neuroradiologists. However, comparing the literature is not a trivial task due to the use of different data sets, each with varying degrees of complexity, suggesting the need for publicly available data sets.

Our study had a few limitations. First, we chose to build the radiomic features-based classifier on imaging data acquired on the same model of MR scanner with acquisitions performed with the same parameters in order to minimize inter-acquisition variability. This choice limited the number of patients included in the study. Several methods are available today to compensate for differences in image quality between scanners (36, 48), which should allow the applicability of our signature in other centers. In addition, no spatial resampling was applied to the MR images prior to feature extraction. Although this step is mandatory to obtain rotationally invariant features, no bias was introduced in the machine learning pipeline, as the entire cohort had exactly the same imaging parameters. The developed signature can finally be generalized to new patients with MR images of different voxel sizes by integrating an additional resampling step [resampling at a voxel size of (0.8 mm × 0.8 mm × 1.2 mm)]. Third, a semi-automatic method was used for tumor delineation and a single radiologist specialized in neurology performed the contouring of the lesions. Perturbation of the contours would have been an alternative to multiple segmentations to evaluate the robustness of the model developed to segmentation (49). However, the semi-automatic contouring process has been shown to be reliable between raters for brain tumors (50). An integrated diagnostic support system should include an automatic segmentation of the volumes of interest to be considered for radiomics analysis. The automation of this step is now possible with high performance as demonstrated by the recent results of the BRATS challenge (51). Then, the radiomic only features-based classifier takes into account imaging data. The addition of the patient’s age, gender, and medical history elements would lead to holistic models enabling to analyze the correlations between radiomic/non-radiomic features, and to better assess the added value of such a signature compared to more readily available clinical features (49). As well, only post-contrast 3DT1 MR images were considered. A more complex classifier combining data from other sequences such as FLAIR, T2 (47) or perfusion MR sequences may improve classification performance. Finally, a larger cohort of lesions studied would enable its generalizability.

In conclusion, we developed a radiomic features-based classifier based on post-contrast 3DT1 MR images that helps in differentiating GBM and solitary metastatic brain tumors with high diagnosis performance. The performance of the radiomic classifier equals that of neuroradiologists however needs to be improved in further studies including feature extraction applied on FLAIR and perfusion sequences. An interesting point is that the radiomic feature with the highest coefficient value in the classifier, namely sphericity, allows an explainability of the developed model. Future studies using this model on larger sets of patients may clarify its role and its benefit in differentiating these two lesions, particularly by a prospective study registered in a trial database.

## Data Availability Statement

The data analyzed in this study is subject to the following licenses/restrictions: Data can be available on demand. Requests to access these datasets should be directed to CR ch.robert@gustaveroussy.fr and ME myriam.edjlali@gmail.com.

## Ethics Statement

The studies involving human participants were reviewed and approved by College de neurochirurgie, Paris. Written informed consent for participation was not required for this study in accordance with the national legislation and the institutional requirements.

## Author Contributions

AdC, AC, ME, SA, SR, and CR designed the research. AdC, AC, ME, SA, SR, and CR performed the research, analyzed, and interpreted the data, and wrote the paper. AT-E and PV reviewed histopathological data. AR, EDez, JP, AT-E, PV, and FD took care of the patients and retrieved the data. AdC, AC, AR, AT-E, SA, EDez, FD, SR, EDeu, CO, PV, JP, ME, and CR revised and approved the paper. All authors contributed to the article and approved the submitted version.

## Funding

This material is based upon work supported by ITMO PhysiCancer, the Fondation pour la Recherche Médicale (FRM; No. DIC20161236437), and Amazon Web Services (AWS). Amazon Web Services was not involved in the study design, collection, analysis, interpretation of data, the writing of this article or the decision to submit it for publication.

## Conflict of Interest

The authors declare that the research was conducted in the absence of any commercial or financial relationships that could be construed as a potential conflict of interest.

The funder was not involved in the study design, collection, analysis, interpretation of data, the writing of this article or the decision to submit it for publication.
