# Supplementary material for: Development of a Machine Learning Classifier Based on Radiomic Features Extracted From Post-Contrast 3D T1-Weighted MR Images to Distinguish Glioblastoma From Solitary Brain Metastasis
Source: Front Oncol. 2021 Jul 13;11:638262. doi: 10.3389/fonc.2021.638262 (PMC8315001; doi:10.3389/fonc.2021.638262)
Supplement: Supplementary file 1 [file Table_1.pdf]

Supplementary Material

Table S1. Grid Search parameters used for each Machine Learning model.

Model	Parameters	Space
AdaBoostClassifier	base_estimator	DecisionTreeClassifier
	max_depth	[1, 2, 3, 4, 5]
	n_estimators	200
	learning_rate	[0.001, 0.01, 0.05, 0.1, 0.25, 0.50, 0.75, 1.0]
BaggingClassifier	base_estimator	DecisionTreeClassifier
	max_depth	[1, 2, 3, 4, 5]
	n_estimators	200
	max_features	[0.2, 0.3, 0.4, 0.5, 0.6, 0.7, 0.8, 0.9, 1.0]
BernoulliNB	alpha	[1e-7, 1e-6, 1e-5, 1e-4, 1e-3, 1e-2, 1e-1, 0.25, 0.50, 0.75, 1.0]
DecisionTreeClassifier	criterion	[”gini”, ”entropy”]
	splitter	[”best”, ”random”]
	max_depth	[10, 20, 30, 40, 50, 60, 70, 80, 90, 100, 110, None]
	min_samples_split	[2, 5, 10]
	min_samples_leaf	[1, 2, 4]
	max_features	[”auto”, ”sqrt”, ”log2”]
	class_weight	[None, ”balanced”]
ExtraTreeClassifier	criterion	[”gini”, ”entropy”]
	splitter	[”best”, ”random”]
	max_depth	[10, 20, 30, 40, 50, 60, 70, 80, 90, 100, 110, None]
	min_samples_split	[2, 5, 10]
	min_samples_leaf	[1, 2, 4]
	max_features	[”auto”, ”sqrt”, ”log2”]
	class_weight	[None, ”balanced”]
ExtraTreesClassifier	n_estimators	200
	criterion	[”gini”, ”entropy”]
	max_depth	[3, 4, 5, 6, 7, 8]
	min_samples_split	[0.005, 0.01, 0.05, 0.10]

continued on next page

Table S1 – *continued from previous page*

Model	Parameters	Space
	min_samples_leaf	[0.005, 0.01, 0.05, 0.10]
	max_features	[”auto”, ”sqrt”, ”log2”]
	class_weight	[None, ”balanced”]
GaussianNB	var_smoothing	[1e-9, 1e-8, 1e-7, 1e-6, 1e-5]
GradientBoostingClassifier	learning_rate	[0.15, 0.1, 0.05, 0.01, 0.005, 0.001]
	n_estimators	200
	subsample	[0.8, 0.9, 1]
	min_samples_split	[0.005, 0.01, 0.05, 0.10]
	min_samples_leaf	[0.005, 0.01, 0.05, 0.10]
	max_depth	[2, 3, 4, 5, 6]
	max_features	[”auto”, ”sqrt”, ”log2”]
KNeighborsClassifier	n_neighbors	[1, 20]
	weights	[”uniform”, ”distance”]
	algorithm	[”auto”, ”ball_tree”, ”kd_tree”, ”brute”]
	leaf_size	[5, 50]
	p	1
LinearSVC	C	[0.0001, 0.001, 0.01, 0.1, 1.0, 10, 100]
	penalty	”l1”
	dual	False
	penalty	”l2”
	dual	[True, False]
LogisticRegression	C	[0.1, 0.3, 0.5, 0.7, 0.9, 1.1, 1.3, 1.5, 1.7, 1.9, 2.1, 2.3, 2.5, 2.7, 2.9]
	fit_intercept	[True, False]
	penalty	”l1”
	solver	”lbfgs”
	penalty	”l2”
	solver	[”lbfgs”, ”liblinear”]
MLPClassifier	hidden_layer_sizes	[(1, 5), (1, 10), (1, 15), (1, 20), (1, 25), (1, 30), (1, 35), (1, 40), (1, 45), (1, 50), (1, 55), (1, 60), (1, 65), (1, 70), (1, 75), (1, 80), (1, 85), (1, 90), (1, 95), (1, 100), (1, 105), (1, 110), (1, 115), (2, 5), (2, 10), (2, 15), (2, 20), (2, 25), (2, 30), (2, 35), (2, 40), (2, 45), (2, 50), (2, 55), (2, 60), (2, 65), (2, 70), (2, 75), (2, 80), (2, 85), (2, 90), (2, 95), (2, 100), (2, 105), (2, 110), (2, 115)]

continued on next page

Table S1 – *continued from previous page*

Model	Parameters	Space
	activation	["tanh", "relu"]
	solver	["lbfgs", "sgd", "adam"]
	alpha	[0.1, 0.001, 0.0001]
	learning_rate	["constant", "invscaling", "adaptive"]
QuadraticDiscriminantAnalysis	reg_param	[0.0, 0.01, 0.02, 0.03, 0.04, 0.05, 0.06, 0.07, 0.08, 0.09, 0.1, 0.11, 0.12, 0.13, 0.14, 0.15, 0.16, 0.17, 0.18, 0.19, 0.2, 0.21, 0.22, 0.23, 0.24, 0.25, 0.26, 0.27, 0.28, 0.29, 0.3, 0.31, 0.32, 0.33, 0.34, 0.35, 0.36, 0.37, 0.38, 0.39, 0.4, 0.41, 0.42, 0.43, 0.44, 0.45, 0.46, 0.47, 0.48, 0.49, 0.5, 0.51, 0.52, 0.53, 0.54, 0.55, 0.56, 0.57, 0.58, 0.59, 0.6, 0.61, 0.62, 0.63, 0.64, 0.65, 0.66, 0.67, 0.68, 0.69, 0.7, 0.71, 0.72, 0.73, 0.74, 0.75, 0.76, 0.77, 0.78, 0.79, 0.8, 0.81, 0.82, 0.83, 0.84, 0.85, 0.86, 0.87, 0.88, 0.89, 0.9, 0.91, 0.92, 0.93, 0.94, 0.95, 0.96, 0.97, 0.98, 0.99, 1.0]
RandomForestClassifier	n_estimators	[3, 11, 20]
	criterion	["gini", "entropy"]
	max_depth	[10, 20, 30, 40, 50, 60, 70, 80, 90, 100, 110, None]
	min_samples_split	[2, 5, 10]
	min_samples_leaf	[1, 2, 4]
	max_features	["auto", "sqrt", "log2"]
	class_weight	[None, "balanced"]
RidgeClassifier	alpha	[1e-7, 1e-6, 1e-5, 1e-4, 1e-3, 1e-2, 1e-1, 0.25, 0.50, 0.75, 1.0]
SGDClassifier	alpha	[1e-7, 1e-6, 1e-5, 1e-4, 1e-3, 1e-2, 1e-1, 0.25, 0.50, 0.75, 1.0]

Notes.
If a parameter is not mentioned, it corresponds to the default value of version 0.23.2 of the scikit-learn library.

Table S2. Ranked performance of all considered associations combining 9 feature scaling methods and 16 classifiers, obtained using a 5x5 nested cross-validation. Ranking was based on the best generalization score, i.e. the mean and standard deviation (Std) of the Balanced Accuracy corresponding to the outer loop.

**Rank**	**Scaler**	**Model**	**Mean outer score**	**Std outer score**
1	PowerTransformer-yeo-johnson	LogisticRegression	0.862	0.041
2	StandardScaler	LinearSVC	0.841	0.045
3	RobustScaler	LogisticRegression	0.835	0.048
4	PowerTransformer-yeo-johnson	LinearSVC	0.834	0.041
5	QuantileTransformer-uniform	RidgeClassifier	0.834	0.054
6	QuantileTransformer-normal	SGDClassifier	0.834	0.073
7	QuantileTransformer-normal	MLPClassifier	0.834	0.083
8	QuantileTransformer-normal	LogisticRegression	0.828	0.076
9	PowerTransformer-yeo-johnson	RidgeClassifier	0.827	0.057
10	StandardScaler	LogisticRegression	0.822	0.087
11	QuantileTransformer-uniform	MLPClassifier	0.814	0.081
12	MaxAbsScaler	RidgeClassifier	0.813	0.071
13	MinMaxScaler	RidgeClassifier	0.811	0.087
14	MinMaxScaler	MLPClassifier	0.806	0.077
15	StandardScaler	MLPClassifier	0.801	0.057
16	PowerTransformer-yeo-johnson	QuadraticDiscriminantAnalysis	0.800	0.037
17	QuantileTransformer-normal	LinearSVC	0.800	0.051
18	RobustScaler	LinearSVC	0.800	0.052
19	QuantileTransformer-uniform	LinearSVC	0.800	0.082
20	PowerTransformer-yeo-johnson	SGDClassifier	0.793	0.046
21	No Scaler	LinearSVC	0.790	0.038
22	PowerTransformer-yeo-johnson	MLPClassifier	0.786	0.056
23	MinMaxScaler	SGDClassifier	0.786	0.058
24	StandardScaler	SGDClassifier	0.785	0.081
25	RobustScaler	RidgeClassifier	0.783	0.055
26	QuantileTransformer-uniform	LogisticRegression	0.780	0.040
27	MinMaxScaler	LogisticRegression	0.778	0.059
28	MinMaxScaler	LinearSVC	0.778	0.070
29	No Scaler	LogisticRegression	0.771	0.048
30	MaxAbsScaler	MLPClassifier	0.770	0.067
31	RobustScaler	SGDClassifier	0.766	0.058
32	StandardScaler	RidgeClassifier	0.764	0.078
33	RobustScaler	MLPClassifier	0.759	0.075
34	StandardScaler	QuadraticDiscriminantAnalysis	0.758	0.034
35	MaxAbsScaler	LogisticRegression	0.758	0.099
36	QuantileTransformer-normal	QuadraticDiscriminantAnalysis	0.758	0.110

continued on next page

Table S2 – *continued from previous page*

Rank	Scaler	Model	Mean outer score	Std outer score
37	No Scaler	BaggingClassifier	0.757	0.082
37	StandardScaler	BaggingClassifier	0.757	0.082
37	MinMaxScaler	BaggingClassifier	0.757	0.082
37	MaxAbsScaler	BaggingClassifier	0.757	0.082
37	RobustScaler	BaggingClassifier	0.757	0.082
38	QuantileTransformer-uniform	QuadraticDiscriminantAnalysis	0.752	0.095
39	No Scaler	QuadraticDiscriminantAnalysis	0.751	0.030
40	QuantileTransformer-normal	GradientBoostingClassifier	0.751	0.094
41	No Scaler	AdaBoostClassifier	0.750	0.104
41	StandardScaler	AdaBoostClassifier	0.750	0.104
41	MinMaxScaler	AdaBoostClassifier	0.750	0.104
41	MaxAbsScaler	AdaBoostClassifier	0.750	0.104
41	RobustScaler	AdaBoostClassifier	0.750	0.104
41	QuantileTransformer-normal	AdaBoostClassifier	0.750	0.104
41	QuantileTransformer-uniform	AdaBoostClassifier	0.750	0.104
42	RobustScaler	QuadraticDiscriminantAnalysis	0.744	0.080
43	No Scaler	GradientBoostingClassifier	0.744	0.087
43	StandardScaler	GradientBoostingClassifier	0.744	0.087
43	MinMaxScaler	GradientBoostingClassifier	0.744	0.087
43	MaxAbsScaler	GradientBoostingClassifier	0.744	0.087
43	RobustScaler	GradientBoostingClassifier	0.744	0.087
43	QuantileTransformer-uniform	GradientBoostingClassifier	0.744	0.087
44	PowerTransformer-yeo-johnson	BaggingClassifier	0.743	0.081
44	PowerTransformer-yeo-johnson	GradientBoostingClassifier	0.743	0.081
45	QuantileTransformer-normal	BaggingClassifier	0.743	0.085
46	StandardScaler	BernoulliNB	0.743	0.111
47	Normalizer	RandomForestClassifier	0.739	0.043
48	MaxAbsScaler	LinearSVC	0.738	0.096
49	QuantileTransformer-uniform	ExtraTreesClassifier	0.737	0.089
50	No Scaler	ExtraTreesClassifier	0.736	0.073
50	StandardScaler	ExtraTreesClassifier	0.736	0.073
50	MinMaxScaler	ExtraTreesClassifier	0.736	0.073
50	MaxAbsScaler	ExtraTreesClassifier	0.736	0.073
50	RobustScaler	ExtraTreesClassifier	0.736	0.073
51	QuantileTransformer-uniform	BaggingClassifier	0.736	0.093
52	QuantileTransformer-normal	RandomForestClassifier	0.736	0.113
52	QuantileTransformer-uniform	RandomForestClassifier	0.736	0.113
53	Normalizer	GradientBoostingClassifier	0.730	0.089
54	No Scaler	RandomForestClassifier	0.730	0.107
54	StandardScaler	RandomForestClassifier	0.730	0.107

continued on next page

Table S2 – *continued from previous page*

Rank	Scaler	Model	Mean outer score	Std outer score
54	MinMaxScaler	RandomForestClassifier	0.730	0.107
54	MaxAbsScaler	RandomForestClassifier	0.730	0.107
54	RobustScaler	RandomForestClassifier	0.730	0.107
55	PowerTransformer-yeo-johnson	AdaBoostClassifier	0.730	0.116
56	MaxAbsScaler	QuadraticDiscriminantAnalysis	0.725	0.078
57	PowerTransformer-yeo-johnson	ExtraTreesClassifier	0.723	0.060
58	RobustScaler	KNeighborsClassifier	0.723	0.073
59	MinMaxScaler	QuadraticDiscriminantAnalysis	0.718	0.066
60	MaxAbsScaler	SGDClassifier	0.717	0.089
61	MaxAbsScaler	KNeighborsClassifier	0.715	0.083
62	StandardScaler	KNeighborsClassifier	0.714	0.085
63	QuantileTransformer-normal	GaussianNB	0.714	0.104
64	Normalizer	BaggingClassifier	0.711	0.076
65	StandardScaler	GaussianNB	0.710	0.037
65	MinMaxScaler	GaussianNB	0.710	0.037
65	MaxAbsScaler	GaussianNB	0.710	0.037
65	RobustScaler	GaussianNB	0.710	0.037
66	QuantileTransformer-uniform	SGDClassifier	0.710	0.096
67	QuantileTransformer-normal	ExtraTreesClassifier	0.708	0.070
68	PowerTransformer-yeo-johnson	RandomForestClassifier	0.708	0.106
69	QuantileTransformer-normal	RidgeClassifier	0.707	0.082
70	MinMaxScaler	KNeighborsClassifier	0.700	0.077
71	Normalizer	ExtraTreesClassifier	0.697	0.061
72	QuantileTransformer-uniform	GaussianNB	0.695	0.104
73	PowerTransformer-yeo-johnson	GaussianNB	0.688	0.096
74	QuantileTransformer-uniform	KNeighborsClassifier	0.687	0.106
75	Normalizer	AdaBoostClassifier	0.682	0.079
76	No Scaler	RidgeClassifier	0.679	0.019
77	QuantileTransformer-uniform	DecisionTreeClassifier	0.673	0.094
77	QuantileTransformer-uniform	ExtraTreeClassifier	0.673	0.094
78	PowerTransformer-yeo-johnson	DecisionTreeClassifier	0.671	0.077
78	PowerTransformer-yeo-johnson	ExtraTreeClassifier	0.671	0.077
79	PowerTransformer-yeo-johnson	BernoulliNB	0.667	0.097
80	No Scaler	DecisionTreeClassifier	0.666	0.178
80	StandardScaler	DecisionTreeClassifier	0.666	0.178
80	MinMaxScaler	DecisionTreeClassifier	0.666	0.178
80	MaxAbsScaler	DecisionTreeClassifier	0.666	0.178
80	RobustScaler	DecisionTreeClassifier	0.666	0.178
80	No Scaler	ExtraTreeClassifier	0.666	0.178
80	StandardScaler	ExtraTreeClassifier	0.666	0.178

continued on next page

Table S2 – *continued from previous page*

Rank	Scaler	Model	Mean outer score	Std outer score
80	MinMaxScaler	ExtraTreeClassifier	0.666	0.178
80	MaxAbsScaler	ExtraTreeClassifier	0.666	0.178
80	RobustScaler	ExtraTreeClassifier	0.666	0.178
81	RobustScaler	BernoulliNB	0.652	0.109
81	QuantileTransformer-normal	BernoulliNB	0.652	0.109
82	PowerTransformer-yeo-johnson	KNeighborsClassifier	0.651	0.096
83	Normalizer	KNeighborsClassifier	0.645	0.089
84	No Scaler	GaussianNB	0.645	0.106
85	Normalizer	RidgeClassifier	0.640	0.073
86	No Scaler	BernoulliNB	0.639	0.098
86	MaxAbsScaler	BernoulliNB	0.639	0.098
86	Normalizer	BernoulliNB	0.639	0.098
87	Normalizer	MLPClassifier	0.626	0.053
88	QuantileTransformer-normal	KNeighborsClassifier	0.624	0.084
89	QuantileTransformer-normal	DecisionTreeClassifier	0.622	0.060
89	QuantileTransformer-normal	ExtraTreeClassifier	0.622	0.060
89	Normalizer	DecisionTreeClassifier	0.610	0.060
89	Normalizer	ExtraTreeClassifier	0.610	0.060
90	Normalizer	QuadraticDiscriminantAnalysis	0.604	0.118
91	No Scaler	KNeighborsClassifier	0.584	0.073
92	Normalizer	LinearSVC	0.570	0.049
93	Normalizer	GaussianNB	0.549	0.035
94	No Scaler	MLPClassifier	0.542	0.051
95	Normalizer	SGDClassifier	0.513	0.017
96	No Scaler	SGDClassifier	0.507	0.014
97	MinMaxScaler	BernoulliNB	0.501	0.041
97	QuantileTransformer-uniform	BernoulliNB	0.501	0.041
98	Normalizer	LogisticRegression	0.499	0.021

Notes.
No Scaler corresponds to the fact of not using a scaler in the machine learning pipeline.
